# Supplementary material for: Iterative improvement in the automatic modular design of robot swarms
Source: PeerJ Comput Sci. 2020 Dec 7;6:e322. doi: 10.7717/peerj-cs.322 (PMC7924708; doi:10.7717/peerj-cs.322)
Supplement: Supplemental Information 3 [file peerj-cs-06-322-s003.zip › argos3/doc/api/standalone/a00487_source.html]

ARGoS: plugins/robots/generic/control\_interface/ci\_altitude\_sensor.h Source File


- Main Page
- Related Pages
- Namespaces
- Classes
- Files

- File List
- File Members

# plugins/robots/generic/control\_interface/ci\_altitude\_sensor.h

Go to the documentation of this file.

```
00001 
00007 #ifndef CCI_ALTITUDE_SENSOR_H
00008 #define CCI_ALTITUDE_SENSOR_H
00009 
00010 /* To avoid dependency problems when including */
00011 namespace argos {
00012    class CCI_AltitudeSensor;
00013 }
00014 
00015 #include <argos3/core/control_interface/ci_sensor.h>
00016 
00017 namespace argos {
00018 
00019    class CCI_AltitudeSensor: public CCI_Sensor {
00020 
00021    public:
00022 
00023       CCI_AltitudeSensor();
00024 
00025       virtual ~CCI_AltitudeSensor() {}
00026 
00030       Real GetReading() const;
00031 
00032 #ifdef ARGOS_WITH_LUA
00033       virtual void CreateLuaState(lua_State* pt_lua_state);
00034       virtual void ReadingsToLuaState(lua_State* pt_lua_state);
00035 #endif
00036 
00037    protected:
00038 
00039       Real m_fReading;
00040 
00041    };
00042 
00043 }
00044 
00045 #endif
```

---

Generated on 10 Jul 2018 for ARGoS by 
 1.6.1
